# Insect Detection and Classification Based on an Improved Convolutional Neural Network

**DOI:** 10.3390/s18124169

**Published:** 2018-11-27

**Authors:** Denan Xia, Peng Chen, Bing Wang, Jun Zhang, Chengjun Xie

**Affiliations:** 1 School of Computer Science and Technology, Anhui University, Hefei 230601, Anhui, China; ahu0086@163.com; 2 Institute of Physical Science and Information Technology, Anhui University, Hefei 230601, Anhui, China; 3 School of Electrical and Information Engineering, Anhui University of Technology, Ma’anshan 243032, Anhui, China; wangbing@ustc.edu; 4 School of Electrical Engineering and Automation, Anhui University, Hefei 230601, Anhui, China; 5 Institute of Intelligent Machines, and Hefei Institutes of Physical Science, Chinese Academy of Sciences, Hefei 230031, Anhui, China; cjxie@iim.ac.cn

**Keywords:** convolutional neural network, insect detection, field crops, region proposal network, VGG19

## Abstract

Regarding the growth of crops, one of the important factors affecting crop yield is insect disasters. Since most insect species are extremely similar, insect detection on field crops, such as rice, soybean and other crops, is more challenging than generic object detection. Presently, distinguishing insects in crop fields mainly relies on manual classification, but this is an extremely time-consuming and expensive process. This work proposes a convolutional neural network model to solve the problem of multi-classification of crop insects. The model can make full use of the advantages of the neural network to comprehensively extract multifaceted insect features. During the regional proposal stage, the Region Proposal Network is adopted rather than a traditional selective search technique to generate a smaller number of proposal windows, which is especially important for improving prediction accuracy and accelerating computations. Experimental results show that the proposed method achieves a heightened accuracy and is superior to the state-of-the-art traditional insect classification algorithms.

## 1. Introduction

Insects are known to be a major factor in the world’s agricultural economy, therefore it is particularly crucial to prevent and control agricultural insects [1], through the use of programs such as dynamic surveys and insect population management by real-time monitoring systems [2]. However, there are many species of insects in farmlands, which requires a lot of time for manual classification by insect experts [3]. It is well known that different species of insects might have similar phenotypes, and insects often take on complicated phenotypes due to different environments and growth periods [4,5]. Since people without the knowledge of entomology cannot distinguish insect categories and the growth period of insects, it is necessary to develop more rapid and effective approaches to tackle this problem.

The development of machine learning algorithms has provided an excellent solution for insect image recognition [6,7,8]. Computer vision [9] and machine learning methods have achieved great successes in vehicle identification and pedestrian detection. Li et al. [10] combined convolutional neural networks (CNN) with an edge boxes algorithm to accurately recognize pedestrians in images. Several issues have to be addressed in the process of the recognition and classification of insects, however, which are briefly described as follows:

1) Quickly locate the information of an insect positioned in a complex background;
2) Accurately distinguish insect species with high similarity between intra-class and inter-class;
3) Effectively identify the different phenotypes of the same insect species in different growth periods.

Xie et al. [7] combined a sparse-coding technique for encoding insect images with a multiple-kernel learning (MKL) technique to construct an insect recognition system, which achieved a mAP (mean average precision) of 85.5% on 24 common insects in crop fields [11]. Xie’s method requires multi-image preprocessing, however, such as image denoising and segmentation [12,13], which expends a lot of time and technical support, so predictions on images without preprocessing might not be satisfactory. Lim et al. adopted Alexnet and Softmax to build an insect classification system, which was optimized by adjusting the network architecture [14]. Yalcin et al. [15] proposed an image-based insect classification method by using four feature extraction methods: Hu moments (Hu), Elliptic Fourier Descriptors (EFD), Radial Distance Functions (RDF) and Local Binary Patterns (LBP), but these images need preprocessing manually, which is undoubtedly very time consuming. Pjd et al. [16] proposed a prototype automated identification system which distinguishes five parasitic wasps by identifying wing structure differences. Mayo and Watson [17] developed an automatic identification system using support vector machines to recognize the images of 774 live moths, without manually specifying the region of interest (ROI). Ding and Taylor [18] proposed a neural network model based on deep learning [19] to classify and count the number of moths and achieved successful results. Moreover, Ding’s work showed that the model can achieve better results under ideal experimental conditions. The quality of images, however, is often affected by sunlight, obstructions, etc. Meanwhile, these vague images might affect the recognition and classification of insects.

Traditional machine learning algorithms have certain limitations in the field of image recognition. Recently, many researchers have found that deep learning takes enormous advantage of feature extraction in images, implemented through the adaptive learning of artificial neurons, which does not affect the process of artificially seeking and extracting suitable features. The authors propose a convolutional neural network model for insect recognition and classification. The work flow of this model is roughly divided into two stages:

1) During the first stage, VGG19 [20] is adopted, which is a deep network consisting of 19 layers to extract high-dimensional features from insect images, as well as RPN, which combines highly abstracted information trained to learn the actual locations of insects in images;
2) During the second stage, the feature maps are reshaped to a uniform size and converted into a one-dimensional vector for insect classification.

## 2. Materials and Methods

### 2.1. Dataset: Data Preprocessing and Augmentation

**Figure 1:**
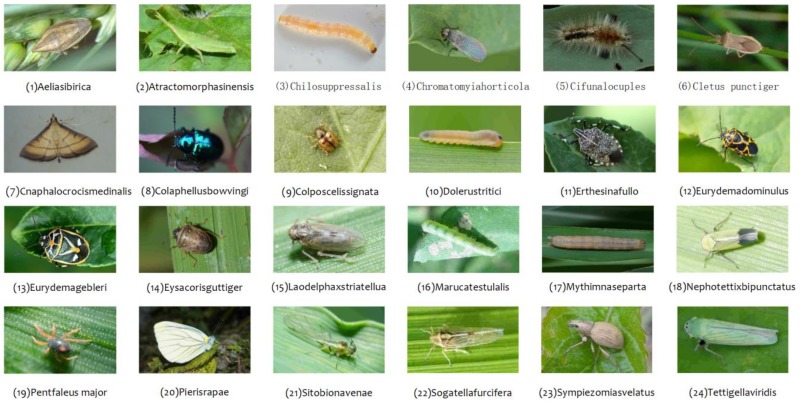
Sample images of 24 insect species collected from crop fields.

The dataset used in Xie’s work [7] was adopted in this work, which contains 24 common images of insects in crop fields, such as *Aelia sibirica*, *Atracto morphasinensis*, *Chilo suppressalis*, etc. Figure 1 shows the scientific names and sample images of 24 insect species. To improve the generalization ability of this model, more images collected from the Internet were used with a data augmentation technique.

Due to the small size of Xie’s data set, collecting new images was required. The authors manually collected images by search engines, such as Baidu and Google, where similar images were extracted manually. Table 1 lists the information of insect species including Xie’s data set and those collected from crop fields and the Internet. Following exclusion of some images with errors and low quality, 660 images were used in this work, where 60 images were randomly selected for the test data set and the remaining 540 images for the training one. 

Moreover, to avoid over-fitting of this model, data augmentation was performed on the training data set to increase the number of training samples. Bilinear interpolation [21] was adopted to fix images to the pixel size of 450 × 750, and all images were then rotated at 90°, 180°, 270° angles. Salt and Pepper Noise [22] was also added to the images to ensure the validity of data, which randomly changes pixel values in the images, whitening some pixel points and blackening some other pixel points. As a result, these techniques expanded the number of training samples to eight times the original ones. Meanwhile, an annotation file containing bounding boxes and the categories of each insect were generated for each image.

Following the data augmentation, the training data set was expanded to 4800 images, where each species of insect included 200 images. The number for the test data set was 480 where each species of insect included 20 images. Thus, the insect data set “MPest” were readied for the next insect identification. 

### 2.2. Deep Learning

Deep learning was proposed by Hinton [19] et al. in 2006, which is a learning model with multiple layers of hidden perceptrons. It combines low-level features with more abstract high-level features to discover estimable relationships in mass data sets [23]. The multi-layer perceptrons are adopted to explore sophisticated structures with multiple abstract levels. The deep convolutional neural network seeks hidden relationships in complex data by using the back-propagation algorithm to adjust the parameters of neurons at each layer. Lecun proposed a multilayer neural network trained with the back-propagation algorithm, which performed at a lower error rate, on data sets of handwritten characters [24]. Deep learning, being better than the state-of-the-art traditional machine learning algorithms, has greatly improved the capabilities of image recognition and more.

### 2.3. Overall CNN Architecture

**Figure 2:**
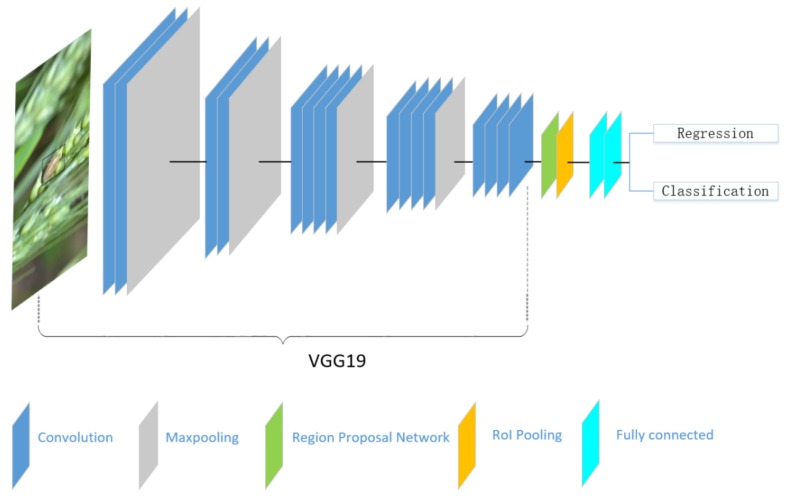
The schematic structure of the proposed detection model based on VGG19.

An improved network architecture was implemented based on VGG19 [20]. Figure 2 shows the schematic diagram of this network. The 19-layer CNN network can be thought of as a self-learning progression of local image features from low to mid to high level. The first 16 convolutional layers of VGG19 were adopted, which were used to extract features. Higher convolutional layers can reduce the resolution of the feature map and extract more abstract high-level features. The Region Proposal Network (RPN) [25,26] was adopted in the first 16 layers, which can recommend the location of insects from a feature map and remove the influence of unrelated background on classification results. Moreover, the last FC6 and FC7 full connection layers were used to capture complex comprehensive feature information. This architecture is appropriate for learning local features from a complex natural image dataset [27]. 

### 2.4. Region Proposal Network

**Figure 3:**
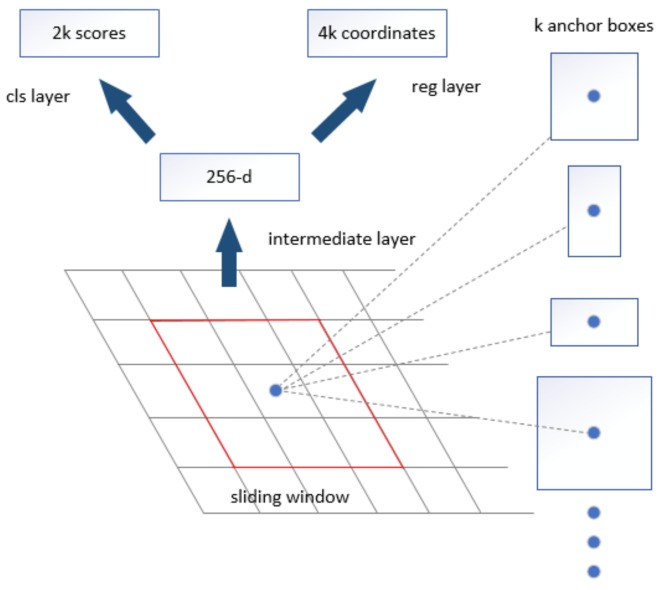
Region Proposal Network (RPN).

Region Proposal Network [25,26] takes an image of arbitrary size as input, and outputs a set of rectangle proposal boxes, where each box has an object score. To generate regional proposals, a small network, which takes an *n × n* spatial window of a convolutional feature map as input, was moved on the convolutional map of the last shared convolutional output. A *3 × 3* sliding window was selected for convolution mapping and generated a 512-dimensional feature vector. Figure 3 shows the architecture of the Region Proposal Network used in this work. At the location of each sliding window, multiple regional proposals were generated on the current sliding window and corresponded to various scales and aspect ratios. Here three scales and three aspect ratios were used, which resulted in *k* = 9 regional proposals for each slide location. The proposals are also called anchors. The regional proposals were then input into two full-connected layers—the bounding box regression layer (*reg*) and the bounding box classification layer (*cls*).

To train the Region Proposal Network, for one image, the target proposal area in the image was assigned a plurality of binary class labels (insects or backgrounds), while the remaining area was discarded. The proposal region was assigned to a positive label, if it had the highest intersection-over-union (IoU) overlap ratio with the ground truth box; otherwise, it was assigned to a negative label if the IoU of the proposal region was lower than the IoU threshold of all ground-truth boxes. The IoU ratio is defined as follows:

(1) $$\mathrm{IoU}=\frac{area(B_{pest}\cap B_{\mathrm{ground}})}{area(B_{pest}\cup B_{\mathrm{ground}})},$$

where *area(B_pest_∩ B_ground_)* represents the intersection area of the insect proposal box and ground truth box, and *area(B_pest_∪* *B_ground_)* denotes their union area. 

Given one image with insects, it is hoped to extract a fixed-length feature vector for an insect from a complex image background by convolution operation. The region of interest (ROI) pooling was adopted to convert insect-like regions into a fixed spatial size which facilitates the generation of the same dimensional feature vectors, because these insect-like regions have different sizes. Then, each ROI feature map was input into the FC6 and FC7 fully connected layers whose output was a 4096-dimension feature vector including the location and category information of the target. The feature vector was then input into the Softmax layer to identify insects and estimate the insect-like regions simultaneously.

#### Training Region Proposal Network

Each input image was resized to 450 × 750 pixels as previously discussed. The feature map was obtained from the input image by the convolution operation of this network. However, convolution operation lead to huge mathematical operations, which might result in the problem of gradient explosion or gradient disappearance. Therefore, it was necessary to add a rectification non-linearities (Relu) layer to activate or suppress the output characteristic diagram of each convolutional layer. A max pooling layer was added after the second, fourth, seventh, eleventh and fifteenth convolutional layers. Moreover, to prevent network over-fitting, the pre-trained VGG19 model was adopted to initialize the first sixteen convolutional layers in this model. 

### 2.5. Loss Function

Regarding loss function of this model, different loss functions were employed for the bounding box regression layer and the bounding box classification layer. The former layer can make an insect-like category score *P* for each predicted region, while the latter one can output a coordinate vector *loc* = (*x*, *y*, *m*, *n*) for each predicted region, where *x* and *y* denote the horizontal and vertical coordinates of the predicted region, respectively, while *m* and *n* denote the width and height of the predicted region. Subsequent to defining the loss functions for classification and regression, they were combined by following Girshick’s multi-task loss rule [28] thus, the whole loss function of this proposed model is:

(2) $$L(\{\alpha_{i}\},\{s_{i}\})=\frac{\sum_{i}L_{cls}(\alpha_{i},\alpha_{{}_{i}}^{*})}{N_{cls}}+\frac{\sum_{i}\alpha_{{}_{i}}^{*}L_{reg}(s_{i},s_{{}_{i}}^{*})}{\beta N_{\mathrm{reg}}},$$

where $\alpha_{{}_{i}}^{}$ is the predicted probability of anchor *i* being an object; the ground-truth label $\alpha_{{}_{i}}^{*}$ is 1 if the region box is positive, otherwise $\alpha_{{}_{i}}^{*}$ = 0; $s_{i}$ denotes the four parameterized coordinates of the predicted bounding box; and $s_{{}_{i}}^{*}$ is the ground-truth box associated with a positive region box. β is the balancing parameter. *N_cls_* is the mini-batch size and *N_reg_* is the number of anchor locations. *L_cls_* is the classification loss function over two classes (object or background), which is written as follows:

(3) $$L_{cls}(\alpha_{i},\alpha_{i}^{*})=Log[\alpha_{i}\alpha_{i}^{*}+(1-\alpha_{i})(1-\alpha_{i}^{*})].$$

To determine the regression loss, *L_reg_* denotes a smooth *L_1_* loss as:

(4) $$\begin{array}{c} L_{reg}(s_{i},s_{{}_{i}}^{*})=f(s_{i},s_{{}_{i}}^{*})\\ \mathrm{where}\;f(x)=\{\begin{array}{cc} 0.5x^{2} & \mathrm{if}|x|<1\\ |x|-0.5 & \mathrm{otherwise} \end{array}. \end{array}$$

### 2.6. Training Overall Model

**Figure 4:**
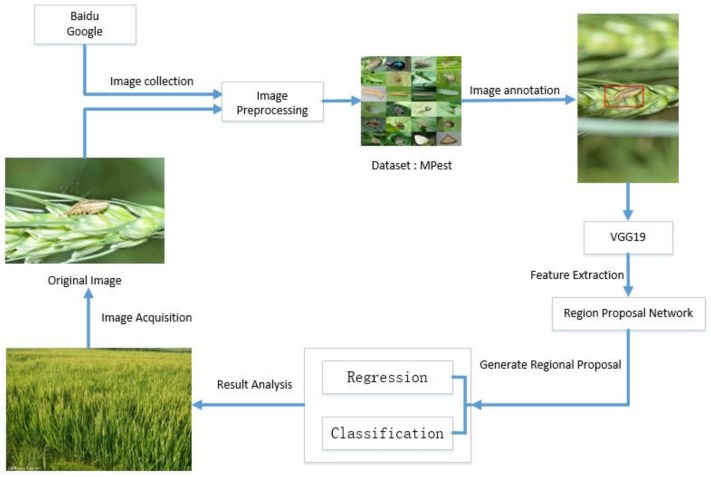
Flowchart of insect recognition and classification. Abundant images were obtained by taking photos in crop fields and collecting images by Baidu and Google online, in which insect images are original and the quality of insect images are uneven. Then, image preprocessing and data augmentation were applied to form our own dataset “MPest”. The model, which was trained on the dataset “MPest”, can effectively help to recognize insects and diseases.

The proposed model was implemented in combination with VGG19 and RPN models. The weights of the network were initialized by the pre-training VGG19 model, while the initial learning rate was 0.001, the momentum was 0.9, and the weight decay was 0.0005. The weights were updated by stochastic gradient descent trick. VGG19 and RPN can be alternately trained to optimize the model rather than training two separate networks. During the first step, the RPN network was trained by learning an image with the location bounding box of insect-like. Next, the predicted region generated by RPN was input to VGG19 to train itself. Finally, both RPN and VGG19 were trained jointly and fine-tuned by fixing shared convolutional network layers. Figure 4 illustrates the flowchart of insect recognition and classification.

## 3. Experiments and Results

This section shows the evaluation of this method for insect recognition and the analysis of this proposed model with different parameters in detail. All experiments were implemented on the framework of Caffe [29] and mean Average Precision (mAP) in Everingham’s work [11] was used as an evaluation metric.

### 3.1. Effects of Feature Extraction Network

**Figure 5:**
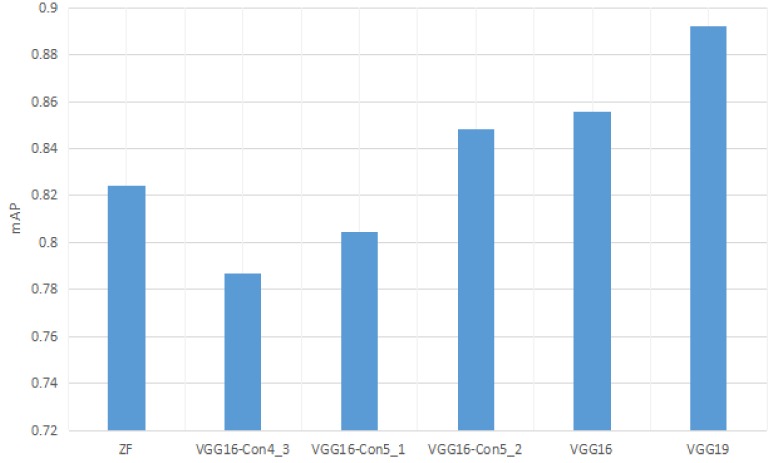
Comparison of different feature extraction methods.

To evaluate the effect of feature extraction on the performance of this model, several feature extraction networks were implemented on the dataset “MPest”, which contains VGG16 with different convolutional features. Generally, the greater number of convolutional layers the model has, the more complex features the model can learn from the images. All of the comparison methods performed 160,000 iterations, with the initialized learning rate of 0.001. Figure 5 shows the performance comparison of the three methods on this dataset. It can be seen that the VGG16 network achieved good performance, but the proposed network achieved an improvement of 3.72% over VGG16. Regarding the ZF network, it consists of a simple architecture of only five convolutional layers and 7 × 7 filters, which results in quick feature map shrink and, thus, cannot extract effectively the multi-faceted features of insects from images. Both VGG16 and VGG19 belong to deep neural networks, where the former contains sixteen convolutional layers and the latter contains nineteen layers. The model with high-level convolution layers can effectively enhance the ability of the proposed model to extract information on insect characteristics under complex backgrounds. Moreover, a 3 × 3 filter was implemented in VGG because multiple 3 × 3 small filters have more nonlinearity than a 7 × 7 large filter, which makes the decision function more decisive. 

**Figure 6:**
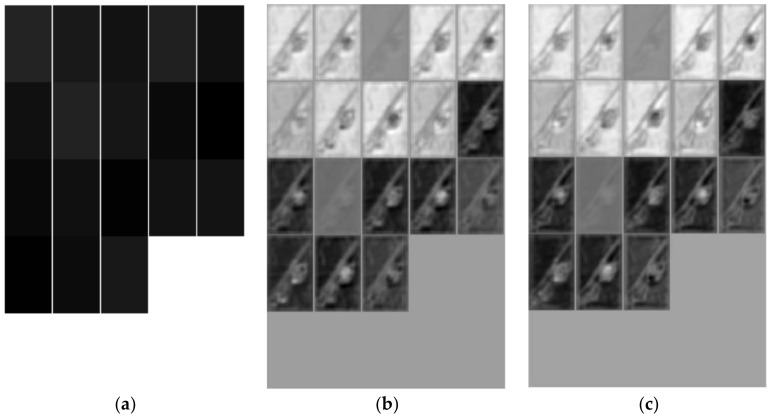
Visualization of feature maps of different feature extraction networks. (**a**): ZF Net; (**b**) VGG16; (**c**) VGG19.

Figure 6 shows the convolution map of three feature extraction networks on our dataset “MPest”. The feature maps of the ZF network have a high loss rate of pixel information and the ZF network blurred images so that it was hard to distinguish insects in images. The feature maps of VGG16 showed that the pixel information of images was retained perfectly by using the small filters. Although the feature maps of VGG19 and VGG16 are almost the same, the more abstract high-dimensional information was retained since more convolutional layers were applied in VGG19.

### 3.2. Effects of Iou Threshold

**Figure 7:**
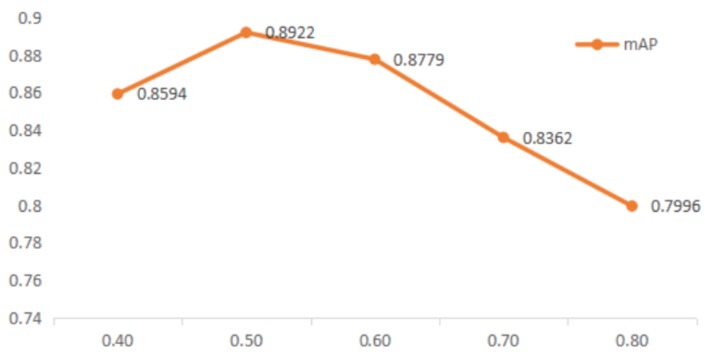
Effects of IoU threshold (VGG19).

The goal of this proposed training task is to reduce the difference between predicted region and ground truth, therefore reducing the influence of unrelated background noise in the predicted region is immensely significant. The IoU threshold score was varied from 0.3 to 0.8 in step sizes of 0.1. Figure 7 shows the mAP curve increased gradually with the IoU threshold, from 0.3 to 0.5. 

That is to say, the increase of the threshold results in abandoning more predicted regions which overlap less with the ground truth. The mAP curve reaches maximum value when the threshold is set to 0.5, where 89.22% of the ground truth are detected successfully. Starting from 0.6 to 0.8, the curve declines slowly. The larger the value of IoU is, the more predicted regions the model abandoned in the regional proposal stage. Therefore, the lower threshold results in an excessively small overlap area between the predicted region and ground truth, where more backgrounds were present in the classification task. Higher thresholds lead to larger discarded prediction regions, which results in an unsuccessful training model.

### 3.3. Effects of Learning Rate

**Figure 8:**
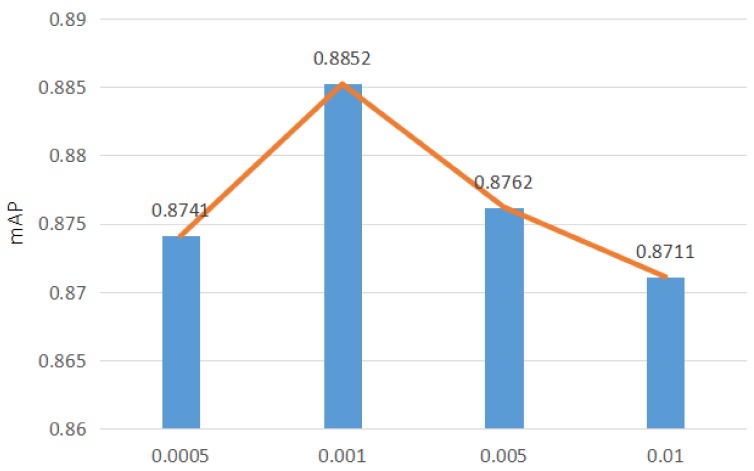
Effects of Learning Rate (VGG19).

Learning rate is an important hyperparameter that controls the update speed of network weights. The effect of the hyperparametric learning rate was investigated from 0.0006 to 0.0014. The learning rate technique of this work is not adaptive. This proposed model first sets an initial learning rate and then decreases it 10 times every 20,000 iterations. Figure 8 shows the mAP of this model with respect to the learning rate.

Looking at Figure 8, it can be seen that, as the learning rate increases, the error of the model gradually decreases. Moreover, a small learning rate leads to a slow update speed of weights of the model and, subsequently, the convergence of the model is not ideal. Therefore, as the learning rate increases, the experimental error of the model gradually decreases. When the learning rate exceeds 0.001, the mAP of the proposed model begins to decrease. A higher learning rate means that the convergence speed of the model is too fast, which results in a larger loss value than expected in the iterative process and makes the model over-fitting.

### 3.4. Performance Comparison with Other Methods

Table 2 shows the performance comparison with other state-of-the-art methods to this study’s test set, such as Single Shot Multibox Detector (SSD) and Fast Region-based Convolutional Neural Network (RCNN). It is known that SSD is a prominent algorithm in which the recurrent feature-pyramid structure is employed to detect images. Several separate predictors were adopted to perform classification and regression tasks at the same time in the multi-feature mapping of the network and the processing of the target detection problem using multi-feature information. The SSD yielded the best performance when it ran 30,000 iterations with a learning rate of 0.001. Obviously, the proposed method outperformed the SSD model and achieved an improvement in mAP of 3.73%. The inference time of the proposed method was the least among the three methods, about 0.083 s. Moreover, the training time of SSD took 38 hours, which was longer than the proposed method.

Fast RCNN is a regional proposal and target classification algorithm that adopts selective search techniques to generate proposal windows. Table 2 shows Fast RCNN achieved a mAP of 0.7964 after 60,000 iterations. However, Fast RCNN took about 70 hours for the training model while the detection time of each image was about 0.195 s. Therefore, fast RCNN requires more computational resources and time for insect detection than this proposed method.

Attempts were made to compare the proposed model with the latest network modules and models. Inception was added to this model, for instance, or different convolution layers of this model were replaced, but experimental results showed that it changed the accuracy of the model little. Moreover, Resnet (Residual Neural Network) could not gain a satisfactory result because the pixel size of images in this work was too large.

Moreover, the differences between this proposed model and other methods can be summarized in two aspects. First, for the insect data set, insect images were collected under field conditions rather than under ideal conditions, which offers the proposed model stronger anti-interference capability. Second, for insect recognition, this proposed method actually can locate insects in images, while most other methods only implemented image classification. To conclude, this proposed method effectively can moderate the distraction of human factors and artificial burdens in processing data set.

## 4. Conclusions and Future Work

A target recognition method based on improved VGG19 for quickly and accurately detecting insects in images was proposed. Since Caffe library provides a pre-trained VGG19 model, whose architecture has achieved a successful balance in feature extraction, the current authors fine-tuned the pre-trained model to train this study’s ideal model. The experimental results on the current dataset “MPest” showed that this method is faster and more accurate than existing methods.

However, there are still some issues in this proposed method, such as target detection error. Therefore, this method performance can be further improved as follows:

1) The insect database needs to be augmented, which can be manually collected in the future;
2) More appropriate models to extract helpful insect-like areas from images should be tried;
3) Regarding the classification task, the classification of insects needs to be more detailed, and the periods of insect growth should be divided. Workers will implement different pest control measures according to the period of insect growth.

## Figures and Tables

**Table 1:** Information of 24 insect species collected from Xie’s data set, crop fields and the Internet.

Species	Quantity	Species	Quantity	Species	Quantity
Aeliasibirica	66	Colposcelissignata	73	Mythimnaseparta	49
Atractomorphasinensis	60	Dolerustritici	91	Nephotettixbipunctatus	66
Chilosuppressalis	53	Erthesinafullo	49	Pentfaleus major	83
Chromatomyiahorticola	51	Eurydemadominulus	128	Pierisrapae	61
Cifunalocuples	47	Eurydemagebleri	42	Sitobionavenae	60
Cletus punctiger	60	Eysacorisguttiger	60	Sogatellafurcifera	71
Cnaphalocrocismedinalis	53	Laodelphaxstriatellua	82	Sympiezomiasvelatus	55
Colaphellusbowvingi	56	Marucatestulalis	56	Tettigellaviridis	55

**Table 2:** Comparison with Other Methods.

Method	mAP	Inference Time(s)/Per Image	Training Time(h)
Proposed method	0.8922	0.083	11.2
SSD	0.8534	0.120	38.4
Fast RCNN	0.7964	0.195	70.1

## Supplementary Materials

The source codes are available online at http://deeplearner.ahu.edu.cn/web/cnnPest.htm.
