# Supplementary material for: Insect Detection and Classification Based on an Improved Convolutional Neural Network
Source: Sensors (Basel). 2018 Nov 27;18(12):4169. doi: 10.3390/s18124169 (PMC6308804; doi:10.3390/s18124169)
Supplement: Supplementary file 1 [file sensors-18-04169-s001.pdf]

We certify that the following article

Insect detection and classification based on improved convolutional neural network

Denan Xia, Peng Chen *, Jun Zhang, Bing Wang *

has undergone English language editing by MDPI. The text has been checked for correct use of grammar and common technical terms, and edited to a level suitable for reporting research in a scholarly journal.
MDPI uses experienced, native English speaking editors. Full details of the editing service can be found at
► www.mdpi.com/authors/english.

MDPI
Multidisciplinary
Digital Publishing
Institute

Basel, November 2018

Martyn Rittman, Ph.D.
English Editing Manager
englishediting@mdpi.com
http://www.mdpi.com/authors/english
